# Identification of HIV gp41-specific antibodies that mediate killing of infected cells

**DOI:** 10.1371/journal.ppat.1007572

**Published:** 2019-02-19

**Authors:** Katherine L. Williams, Megan Stumpf, Nicole Elise Naiman, Shilei Ding, Meghan Garrett, Theodore Gobillot, Dani Vézina, Katharine Dusenbury, Nitya S. Ramadoss, Ryan Basom, Peter S. Kim, Andrés Finzi, Julie Overbaugh

**Affiliations:** 1 Division of Human Biology, Fred Hutchinson Cancer Research Center, Seattle WA, United States of America; 2 Molecular and Cellular Biology Graduate Program, University of Washington and Fred Hutchinson Cancer Research Center, Seattle, WA United States of America; 3 Medical Scientist Training Program, University of Washington, Seattle WA, United States of America; 4 Department of Microbiology, Infectious Diseases and Immunology, Université de Montréal, Montreal, QC, Canada; 5 Divisions of Basic Sciences and Computational Biology, Fred Hutchinson Cancer Research Center, Seattle, WA, United States of America; 6 Department of Genome Sciences, University of Washington, Seattle, WA, United States of America; 7 Stanford ChEM-H and Department of Biochemistry, Stanford University, Stanford, CA, United States of America; 8 Genomics and Bioinformatics Shared Resource, Fred Hutchinson Cancer Research Center, Seattle, WA, United States of America; 9 Chan Zuckerberg Biohub, San Francisco, CA, United States of America; National Institutes of Health-NIAID, UNITED STATES

## Abstract

Antibodies that mediate killing of HIV-infected cells through antibody-dependent cellular cytotoxicity (ADCC) have been implicated in protection from HIV infection and disease progression. Despite these observations, these types of HIV antibodies are understudied compared to neutralizing antibodies. Here we describe four monoclonal antibodies (mAbs) obtained from one individual that target the HIV transmembrane protein, gp41, and mediate ADCC activity. These four mAbs arose from independent B cell lineages suggesting that in this individual, multiple B cell responses were induced by the gp41 antigen. Competition and phage peptide display mapping experiments suggested that two of the mAbs target epitopes in the cysteine loop that are highly conserved and a common target of HIV gp41-specific antibodies. The amino acid sequences that bind these mAbs are overlapping but distinct. The two other mAbs were competed by mAbs that target the C-terminal heptad repeat (CHR) and the fusion peptide proximal region (FPPR) and appear to both target a similar unique conformational epitope. These gp41-specific mAbs mediated killing of infected cells that express high levels of Env due to either pre-treatment with interferon or deletion of *vpu* to increase levels of BST-2/Tetherin. They also mediate killing of target cells coated with various forms of the gp41 protein, including full-length gp41, gp41 ectodomain or a mimetic of the gp41 stump. Unlike many ADCC mAbs that target HIV gp120, these gp41-mAbs are not dependent on Env structural changes associated with membrane-bound CD4 interaction. Overall, the characterization of these four new mAbs that target gp41 and mediate ADCC provides evidence for diverse gp41 B cell lineages with overlapping but distinct epitopes within an individual. Such antibodies that can target various forms of envelope protein could represent a common response to a relatively conserved HIV epitope for a vaccine.

## Author summary

Anti-HIV antibodies can mediate activity by neutralizing cell-free virus, or binding to infected cells and driving antibody-dependent cellular cytotoxicity (ADCC). While numerous discovery efforts have identified and characterized neutralizing antibodies, much less is known about antibodies that mediate ADCC. Here we describe four new antibodies that target the gp41 transmembrane protein of the HIV envelope. Competition experiments and peptide mapping studies together helped narrow down the binding sites for the four antibodies to two conserved regions of the protein. One pair of antibodies targets a common epitope of gp41 while the other pair binds to a more complex discontinuous epitope. *In vitro* activity assays indicated that this second pair of antibodies could drive killing against cells coated with various forms of gp41, and both pairs of antibodies could drive killing of HIV-infected cells. Inducing these types of antibodies following vaccination may represent a more straightforward path to generating a consistent, functional response to a more conserved portion of the HIV envelope protein.

## Introduction

Eliciting an antibody response to the HIV Envelope protein is thought to be the most likely path to an effective vaccine, and there is evidence that both neutralizing and non-neutralizing HIV-specific antibodies can contribute to protection. Indeed, the only HIV vaccine trial to demonstrate measurable protection from HIV infection implicated non-neutralizing antibodies capable of mediating antibody-dependent cellular cytotoxicity (ADCC) [1]. Studies of mother-infant HIV transmission, a setting where both maternal antibodies and antibodies passively acquired by infants *in utero* are present during the period of transmission risk, have similarly implicated ADCC antibodies in protection. Specifically, ADCC-mediating antibodies isolated from breastmilk were correlated with infant infection outcome in women with high viral load [2], and passively acquired ADCC-mediating antibodies correlated with clinical outcome in infants who acquired HIV after birth [3]. Evidence from studies in non-human primate models have similarly supported a role for non-neutralizing ADCC-mediating antibodies in limiting disease pathogenesis [4–17], and antibodies defective in Fc-receptor binding demonstrated reduced protective efficacy [18, 19]. Further investigation into the epitope targets of ADCC-mediating mAbs and their contribution to protection may help inform future vaccine strategies.

Most studies have focused on antibodies directed to gp120, the extracellular Env glycoprotein. The envelope transmembrane protein, gp41, which is required for viral entry, is also a target of both neutralizing and non-neutralizing HIV antibodies [20–24]. During the entry process, gp41 undergoes a series of conformational changes that drive viral and host cell membrane fusion, resulting in opportunities for antibodies to recognize different gp41 epitopes at various stages in the process. Gp41 encodes several key functional domains in its extracellular portion (ectodomain) where antibodies target. These include the fusion peptide, which becomes exposed as a result of structural changes that promote fusion. There are also two heptad repeat (HR) regions (N terminal HR/NHR and C terminal HR/CHR) that are separated by a disulfide-bonded loop (C-C’ loop), which presents an immunodominant epitope. The interaction of the NHR and CHR during the entry process leads to a six-helix bundle structure that joins the viral and cell membranes together. The region at the C-terminus of the extracellular domain of gp41, the membrane proximal region (MPER), is a target of several broadly neutralizing antibodies [22, 24]. Because the extracellular regions of gp41 are conserved, gp41 is an excellent target for cross-reactive antibodies recognizing diverse viral strains [25]. Further, as virus buds from infected cells, some gp120 proteins are shed. As a result, gp41 stumps are exposed on the cell surface [26] and can be targeted by gp41-specific, ADCC-mediating antibodies [13, 23, 27–31].

Env gp41–directed antibodies arise early in infection [32] and several common targets have been described, including antibodies that recognize the C-C’ loop, which encodes an immunodominant epitope of gp41 (referred to as cluster I antibodies) and others that recognize the CHR (cluster II antibodies), with cluster I being common in chronic infection [21, 33–40] and associated with a broad response [39, 41–43]. Anti-cluster I antibodies inhibit HIV via a variety of mechanisms [8, 13, 39, 44–49], including neutralization and ADCC [13, 22, 24, 28, 31, 33, 38, 39], though gp41-specific ADCC-mediating antibodies have been less well studied than neutralizing antibodies. However, there is evidence that ADCC antibodies could provide protection in both model systems and humans. IgA gp41-targeting antibodies have been isolated from highly exposed, HIV-negative individuals [50–53] and associated with protection [54–57]. Moreover, a gp41-based antigen elicited protection in a macaque model of mucosal infection [58]. Studies investigating the anti-viral effects of passively administered ADCC-mediating antibodies, while few relative to the plethora of passive neutralizing antibody studies, also provide some evidence for a non-sterilizing protective effect of gp41 antibodies [8, 12, 39, 45, 59–64], and in particular, an effect of cluster I ADCC-mediating antibodies on viral load [13].

We recently isolated monoclonal antibodies (mAbs) from a clade A-infected individual by selecting B cells that bound to HIV virus-like particles (VLPs) [65]. While some of the reconstructed mAbs recognized gp120, others did not, even though they showed detectable binding to the VLPs used as bait. One such antibody showed evidence of antibody-dependent cellular viral inhibition (ADCVI) activity [65], prompting us to further evaluate the HIV-specific mAbs from this individual that did not recognize gp120. Here we show that several of the VLP-specific antibodies target gp41 and mediate ADCC, including the antibody that demonstrated ADCVI activity. The four mAbs identified in this one individual all arose from independent B cell lineages and target either the immunodominant epitope that defines cluster I or a discontinuous epitope. We used a unique phage display approach to more finely map the epitopes of the two gp41 cluster I antibodies and showed that they have overlapping but distinct epitopes. The two other mAbs both target a similar discontinuous conformational epitope that includes both the CHR and the FPPR portions of gp41. These mAbs also recognize a structure that mimics gp41 stumps and drive ADCC activity against cells coated with this gp41 mimetic.

## Results

### Binding Antibody Multiplex Assay (BAMA) determines specificity for gp41

We previously described twelve antibodies from a clade A HIV-infected individual, QA255, that bound HIV clade A VLPs. One mAb (QA255.187) demonstrated modest neutralization activity. Three mAbs, QA255.105, QA255.157 and QA255.253, mediated ADCC and ADCVI activity; QA255.105 also neutralized HIV [65]. The remaining eight mAbs bound the VLP but did not mediate activity in neutralization or in ADCC assays using gp120 as a target. Unexpectedly, QA255.006 showed ADCVI activity when included as a negative control mAb in that assay despite the fact that it did not mediate ADCC against gp120-coated cells.

**Fig 1:**
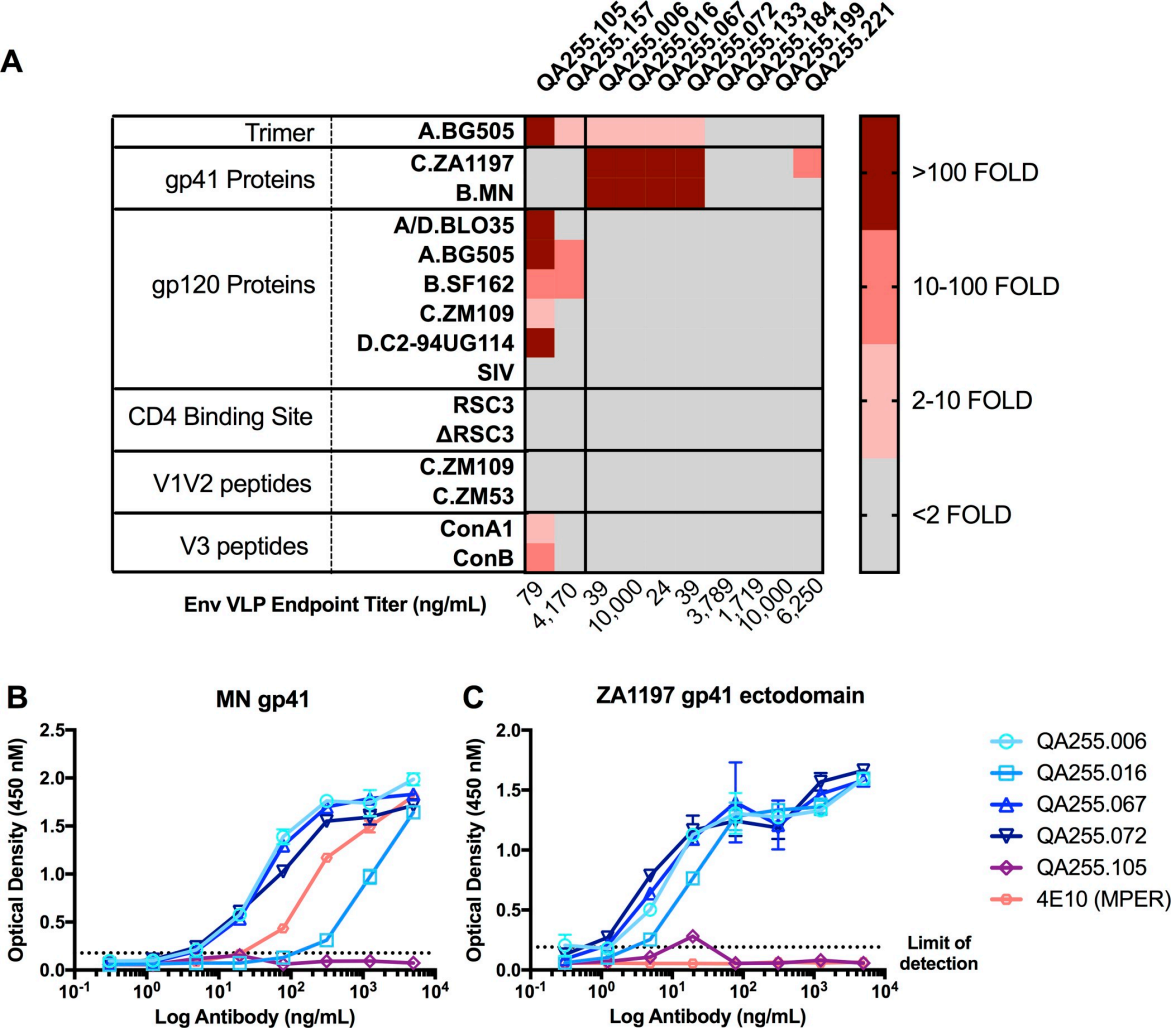
Results of BAMA assay for QA255 antibodies. (A) The binding of the QA255 mAbs indicated at the top of each column are shown in relation to the antigen tested in the BAMA assay, which is indicated in the first two columns. The first two mAbs bind to different epitopes on gp120 [65] and served as controls. Binding to each antigen is defined by the fold increase over background (results with HIV negative plasma) and the binding is color coded as indicated to the right, with increasing shades of red indicating more binding. Gray indicates binding was not detected above background (<2-fold). (B & C) Binding defined by ELISA to MN gp41 and ZA.1197 gp41 ectodomain proteins. Mean absorbance +/- SD is shown in relation to antibody concentration; the dotted line indicates the limit of detection. Data are representative of at least two independent experiments. The key for the mAbs tested is shown to the right.

To explore the epitope specificity and function of these eight antibodies, a Binding Antibody Multiplex Assay (BAMA) that included a panel of 15 antigens was used, with two gp120-specific mAbs from QA255 serving as controls. Each antigen was individually coupled to fluorescent Luminex beads, including two gp41 proteins, five gp120 proteins representing four HIV clades and SIV, a CD4-binding site protein and negative scaffold protein, two clade C V1-V2 peptides, two V3 peptides, and BG505 SOSIP trimer (Fig 1A). Consistent with previous findings that QA255.105 targets V3 [65], this mAb bound to all five HIV gp120 proteins, both V3 peptides and the BG505 trimer. QA255.157, which targets a CD4-induced (CD4i) epitope, bound to two of the five HIV gp120 and the BG505 SOSIP trimer. Of the eight mAbs with unknown epitopes, three did not show detectable binding to any of the proteins tested and one (QA255.221) bound to only one antigen, the gp41 ectodomain at levels just above background. Four antibodies, QA255.006, QA255.016, QA255.067 and QA255.072 bound with a range of 628- to 656-fold above background and 272- to 292-fold above background to the C.ZA.1197 gp41 ectodomain and MN gp41 proteins, respectively, suggesting that these antibodies target the gp41 portion of the HIV trimer (Fig 1A). The very weak binding of these mAbs to the BG505 SOSIP is consistent with prior studies suggesting gp41 epitopes are largely occluded on this soluble form of the trimer [66].

Specificity for gp41 was confirmed by ELISA. QA255.006, QA255.067 and QA255.072 all bound to MN gp41 protein at similar levels (endpoint titer of 4.9 ng/mL), while QA255.016 displayed a less potent endpoint titer of 312.5 ng/mL. MPER-specific mAb 4E10 demonstrated intermediate binding with an endpoint titer of 78.1 ng/mL (Fig 1B). All four antibodies demonstrated comparable binding against C.ZA.1197 ectodomain protein (endpoint titer of 4.9 ng/mL) while the MPER-specific mAb 4E10 was unable to bind the ectodomain protein at any concentration tested, consistent with the absence of MPER in this peptide (Fig 1C). The V3-specific antibody QA255.105 did not demonstrate binding against either of the proteins at any concentration tested (Fig 1B and 1C).

### gp41-specific antibodies demonstrate ADCC activity in the RF-ADCC assay

We next tested whether any of the four gp41-specific antibodies could mediate ADCC activity in the RF-ADCC assay [67], which has shown an association with improved HIV outcomes [2, 3]. Historically, this assay has used target cells coated with gp120 protein. Given that the four QA255 mAbs targeted gp41, we chose to instead coat target cells with the gp41 proteins used in the initial ELISA assays as well as a clade A gp140 protein, which included both gp120 and the extracellular portion of gp41.

**Fig 2:**
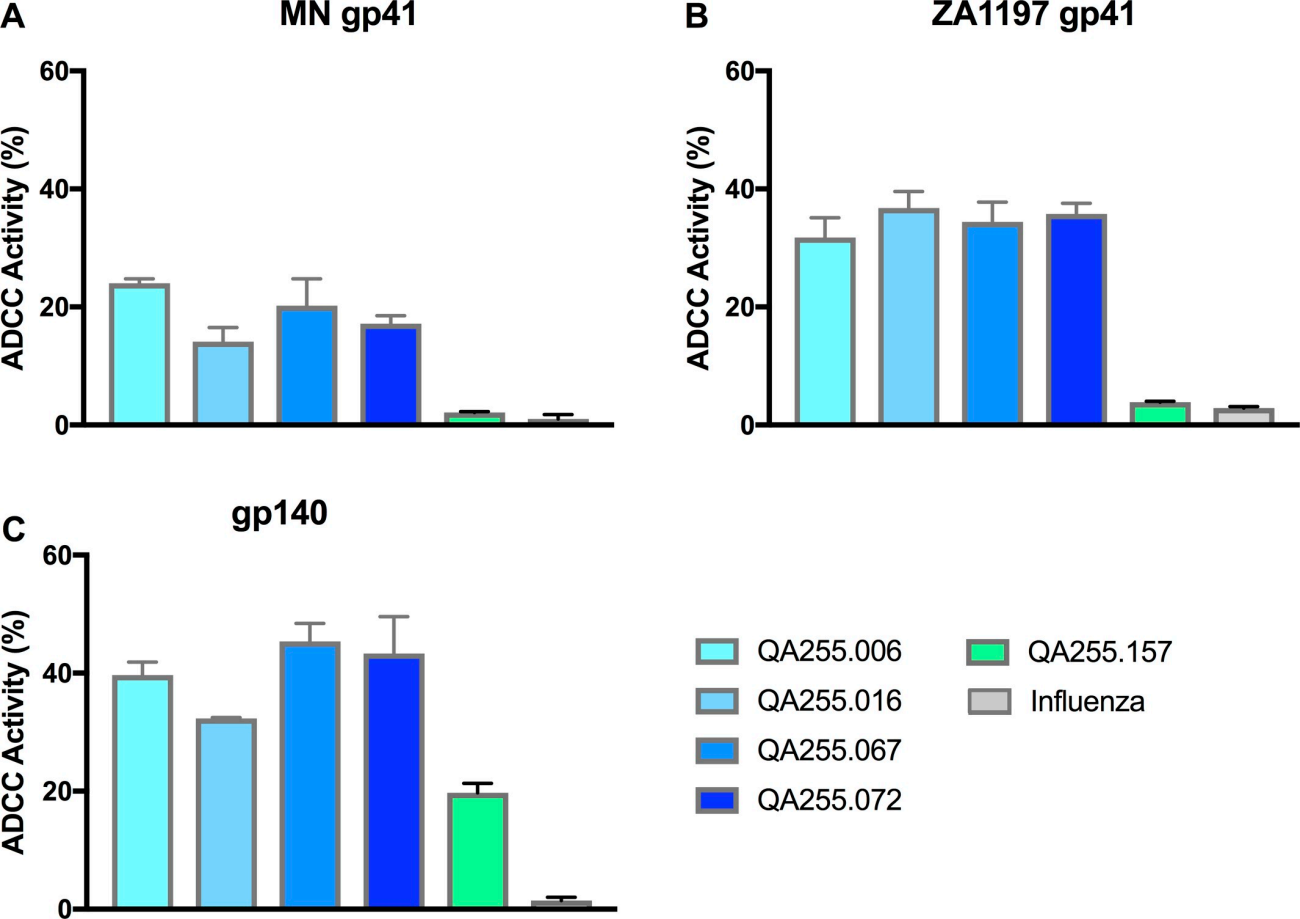
gp41-specific QA255 mAbs mediate ADCC activity. Percent ADCC activity for target cells coated with either (A) clade B MN gp41 protein, (B) clade C ZA.1197 gp41 ectodomain or (C) clade A Q461.e2 TAIV gp140 protein is shown on the y-axis. The key to the mAbs tested is shown in the lower right corner, with QA255 gp41-specific mAbs shown by blue bars, a gp120-specific mAb by green bars, and a control Influenza mAb Fi6_v3 in grey. Results are the average of two replicates +/- SD. The results are representative of studies with PBMCs from two different donors (S1 Fig).

All four gp41-specific mAbs mediated robust activity against cells coated with gp140 and gp41. The four QA255 gp41-specific mAbs demonstrated between 14% - 24% activity against MN gp41 and 32% - 37% activity against C.ZA.1197 gp41 (Fig 2A and 2B). The percent ADCC activity for these four mAbs ranged from 32% - 45% for cells coated with gp140, levels which were slightly higher than gp120-specific control mAb QA255.157. When tested against either of the gp41 proteins, neither QA255.157 nor an influenza-specific mAb, Fi6_v3 mediated measurable activity (Fig 2A–2C), as expected. Similar results were observed with PBMCs from a second donor, although the magnitude of the activity was lower (S1 Fig).

### Competition ELISAs define epitope specificity for gp41-specific QA255 Abs

**Fig 3:**
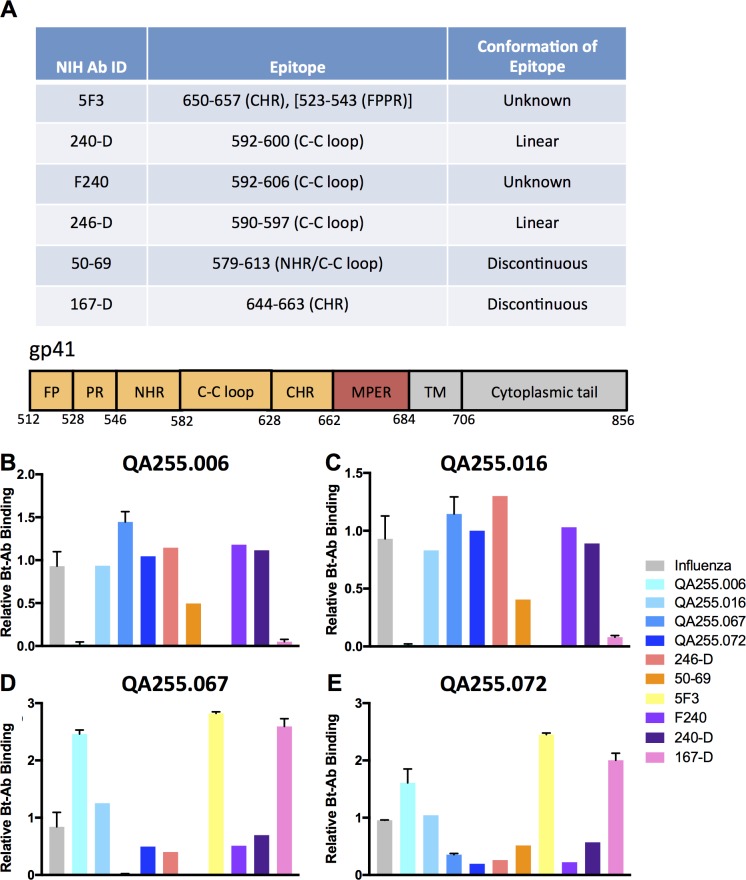
Results of competition ELISAs with mAbs that target known epitopes in gp41. **(**A) The mAbs used for competition experiments and their epitope targets are shown with a schematic of gp41 below. (B-E) Results of competition experiments reported as biotinylated mAb (B) QA255.006, (C) QA255.016, (D) QA255.067, (E) QA255.072 binding in the presence versus absence of the competitor mAbs. The tested Bt-Ab is indicated at the top of each panel, and the competitor mAb is defined by separate colors as specified in the legend to the right of the graphs. The results shown represent the mean relative binding (+/- SD), are from technical duplicates in the same experiment and are representative of at least two biological replicates.

To begin mapping the epitope within the gp41 protein, we tested biotinylated variants of each of the four antibodies in competition with a panel of well-characterized gp41-specific antibodies that target distinct nucleotide residues spanning the ectodomain of the gp41 protein (Fig 3A). Because all four QA255 mAbs bound with comparable efficiency to both the full gp41 protein and the C.ZA.1197 ectodomain variant of gp41 (Fig 1B and 1C), this suggested MPER was not the epitope target and we did not include MPER-targeting antibodies in the competition ELISA. Endpoint ELISAs were performed to confirm binding for the selected six competitor mAbs against the MN gp41 protein. Five of the six mAbs bound with comparable endpoint titers between 4.9–19.5 ng/mL, while mAb 240-D demonstrated a higher endpoint titer of 78.1 ng/mL (S2 Fig).

#### QA255.006

The non-biotinylated version of QA255.006 reduced MN gp41 binding by the autologous biotinylated (BT) variant by 98%, however binding was not affected by the pre-incubation of the other three QA255 gp41-specific mAbs or the Influenza-specific mAb, Fi6_v3. Two mAbs, 167-D, which targets a series of discontinuous residues within CHR [40] and 5F3, which targets the CHR and also has been suggested to interact with the fusion proximal peptide region (FPPR) [34, 68], completely inhibited binding of BT-QA255.006 (95%-100%, respectively) (Fig 3B). MAb 50–69, which targets a discontinuous epitope mapped to the NHR and the C-C’ loop [40], reduced binding of BT-QA255.006 by 51%. Overall, these patterns suggest that QA255.006 targets a discontinuous epitope that includes the CHR and possibly the FPPR and/or portions of the NHR.

#### QA255.016

QA255.016 only demonstrated a modest (17%) inhibition of the biotinylated, autologous variant, whereas QA255.006 reduced BT-QA255.016 binding by 99%. Similar to the QA255.006 results, both mAbs 5F3 and 167-D strongly inhibited Bt-QA255.016 binding and mAb 50–69 partially inhibited binding by 60% (Fig 3C). Neither QA255.067, QA255.072 nor Fi6_v3 inhibited BT-QA255.016. Thus, QA255.016 and QA255.006 appear to target a similar epitope despite originating from independent B cell progenitors (Table 1). Consistent with differences observed in ELISA endpoint titer, QA255.016 showed a more limited ability to compete in this assay as compared to QA255.006 (Fig 1B). These data were consistent with experiments conducted using ZA.1197 ectodomain protein in place of MN gp41, with one relevant difference. When MN gp41 protein was replaced with ZA.1197 ectodomain, both QA255.006 and QA255.016 partially inhibited binding of BT-QA255.016 (S3 Fig).

**Table 1:** Sequence characteristics of QA255 Abs.

	V_H_ gene	V_H_ mut freq (nt, %)	J_H_ gene	D_H_ gene	V_L_ gene	V_L_ mut freq (nt, %)	J_L_ gene
**QA255.006**	**V3-23**	**6.6%**	**J4**	**D2-8**	**LV2-11**	**5.6%**	**J3**
**QA255.016**	**V4-34**	**11.9%**	**J1**	**D2-15**	**LV1-51**	**8.8%**	**J3**
**QA255.067**	**V1-69**	**10.8%**	**J6**	**D5-18**	**LV2-11**	**3.5%**	**J3**
**QA255.072**	**V1-69**	**13.2%**	**J3**	**D3-22**	**KV1-27**	**9.0%**	**J1**

#### QA255.067

QA255.067 completely inhibited binding of the biotinylated, autologous variant and reduced QA255.072 binding by 51%. Pre-incubation with mAb 50–69 completely eliminated BT-QA255.067 binding, while mAbs 246-D, F240 and to a lesser extent 240-D, which all target different residues along the C-C’ loop with either linear or conformational specificity [40, 42] inhibited binding by 60%, 49% and 31%, respectively. Interestingly, pre-incubation with QA255.006, or with 5F3 or 167-D, mAbs previously shown to inhibit QA255.006 binding, increased binding over background levels measured in the absence of a competitor, suggesting that pre-incubation with these mAbs may enhance subsequent binding of QA255.067 (Fig 3D).

#### QA255.072

Pre-incubation of the MN gp41 protein with either autologous QA255.072 or QA255.067 reduced BT-QA255.072 binding by comparable amounts (81% and 65%, respectively), thus suggesting that QA255.067 and QA255.072 target similar epitopes, despite also originating from independent B cell lineages (Table 1). Further, comparison between QA255.067 and QA255.072 inhibition profiles resulted in a strikingly similar pattern. As expected, pre-incubation with QA255.016 or Fi6_v3 did not inhibit QA255.072 binding, while mAbs 246-D, F240 and 240-D all reduced BT-QA255.072 binding to a degree comparable to QA255.067. The greatest deviation between the QA255.067 and QA255.072 binding properties was observed in competition with mAb 50–69, which completely eliminated QA255.067 binding, but reduced QA255.072 binding by only 49%. MAbs 5F3, 167-D and to a lesser extent, QA255.006 all appeared to exacerbate binding activity between 1.6 and 2.5-fold, consistent with observations made with QA255.067 (Fig 3E).

### Phage peptide display identifies specific residues important for QA255.067 and QA255.072 binding

**Fig 4:**
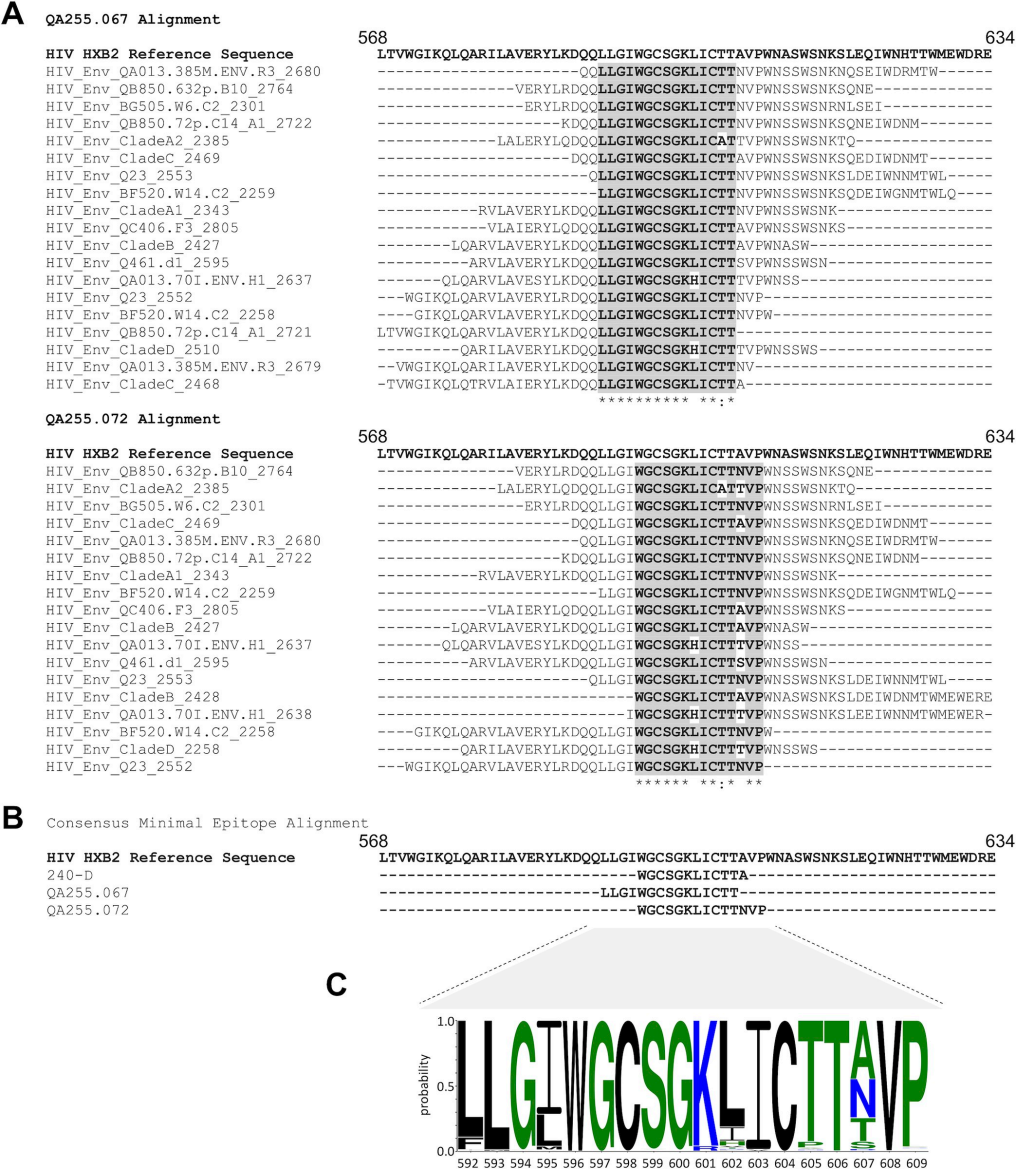
Peptides enriched in phage display immunoprecipitation with gp41 mAbs and their variation in natural sequences. (A) The top two panels show the peptides that were enriched by phage display immunoprecipitation for QA255.067 and QA255.072, with the mostly highly enriched peptides shown at the top of the list. The common sequences among all the enriched peptides are highlighted in gray. (B) This third panel shows a summary of the core sequences identified for these mAbs and compared to 240-D. (C) Logo plot of 5,471 sequences of HIV from the LANL database across the epitopes defined for these mAbs. Colors indicate relative hydrophobicity.

In order to more precisely map the epitopes of these mAbs, we designed a phage immunoprecipitation sequencing approach [69] and determined which peptides in the phage library bound to the QA255 gp41-specific mAbs. A previously defined gp41-specific mAb, 240-D, was tested for comparison [40]. The library contains multiple HIV Env sequences, including consensus sequences for clades A, B, C and D and specific sequences circulating in Kenya. MAb 240-D as well as QA255.067 and QA255.072 all showed enrichment of gp41 peptides from the phage library that encoded sequences from the C-C’ loop and surrounding region, consistent with the predictions from the competition experiments. Sequences that were enriched by binding to mAb QA255.067 shared a common core sequence from 592 to 606 (based on HXB2 numbering), suggesting these amino acids are key parts of the epitope for this mAb (Fig 4A). QA255.072 binding enriched for an overlapping but distinct peptide region that had a common core sequence of amino acids 596 to 609 (Fig 4A). The peptides that were enriched by mAb 240-D were also similar but distinct from the QA255 mAbs and encompassed amino acids 596 to 605 (Fig 4B), which is consistent with the known epitope originally defined by linear peptide ELISA as including 579 to 604 [40, 70]. All HIV strains present in the phage library were represented amongst the significant hits for 240-D, QA255.067, and QA255.072. No non-Env peptides were present in the top 99^th^ percentile of enriched peptides from 240-D, QA255.067, or QA255.072 when ranked by–log 10 p-value. MAbs QA255.006 and QA255.016 did not enrich for any peptides present in the phage library.

Fig 4C shows a logo plot of circulating HIV sequences in the region of gp41 targeted by these mAbs indicating that the epitope target is highly conserved. In the case of QA255.067, the epitope appears to exclude the most variable amino acid in this region at position 607, although it includes the variable position 595. By contrast, QA255.072 excludes the variable position at 595 but includes the variable 607 amino acid. Interestingly, the results from phage display suggest that the QA255.072 mAb tolerates variability at position 607 as peptides with a variety of amino acids at that position are enriched. Overall, these data suggest that the QA255 gp41-specific mAbs should recognize diverse strains of HIV from different clades.

Interestingly, longitudinal sequences from QA255, starting at 21 days post-infection, show no variation within the C-C’ loop epitope of QA255.067 and QA255.072 over time (S4 Fig), perhaps reflecting the highly conserved nature of this domain. The epitopes for the mAbs QA255.006 and QA255.016 were defined only based on competition experiments with other mAbs. When we examined the epitope of these competing mAbs (5F3 and 167-D), which are focused on the CHR and potentially the fusion peptide [34, 40, 68] we see some evidence of variation in those regions (S4 Fig). However, because we have not finely mapped the QA255.006 and QA255.016 epitopes, we cannot say with certainty these residues are included within the actual epitope and represent escape variants.

### QA255.006 and QA255.016 mediate ADCC activity against a post-fusion gp41 stump mimetic

Following interaction of gp120 with CD4 and CCR5 on the surface of target cells, the gp120-gp41 complex undergoes a series of conformational rearrangements, including initial formation of a pre-hairpin fusion intermediate for virus-cell fusion followed by rearrangement into a post-fusion stable six-helix bundle. To determine whether any of the four gp41-specific antibodies could bind to, or mediate functional activity against either the pre- or post-fusion gp41 intermediates, we tested the antibodies against two gp41 mimetic structures [71] including: 1) a 6-Helix structure that is a six alpha-helix bundle forming a hairpin trimer, and likely forms a post-fusion conformation displayed on the surface of infected cells and 2) a 5-Helix structure that is similar to the 6-Helix, but does not contain one C-peptide and presumably acts by inhibiting membrane fusion.

**Fig 5:**
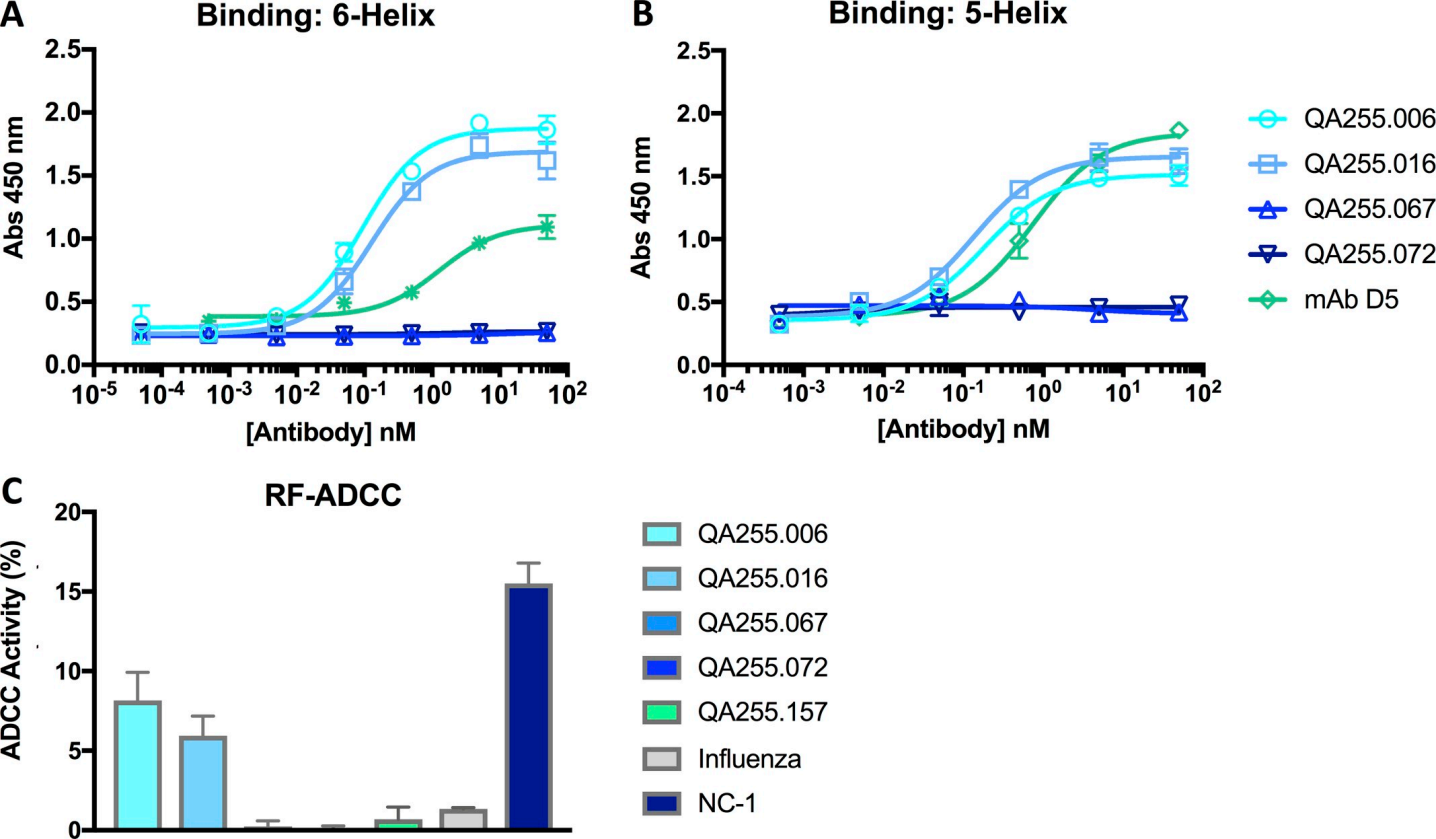
Binding to and ADCC activity against pre- and post-fusion gp41 intermediates. (A-B) Average binding (+/- SD) to the (A) 6-Helix and (B) 5-Helix peptide is measured on the y-axis. Monoclonal antibody D5, which binds to the NHR region, was used as a positive control. (C) ADCC activity is measured against the 6-Helix protein using a modified version of the RF-ADCC assay. Average ADCC activity (+/- SD) is shown for each of the mAbs indicated by the key on the right. Data are representative of at least three independent experiments.

When tested for binding activity, QA255.006 and QA255.016 bound similarly to the 6-Helix and 5-Helix trimers, with EC_50_ values calculated between 97–135 pM. In contrast, control mAb D5, which binds to the NHR and was isolated from a HIV-naïve human scFv phage-display library [72], bound measurably, but poorly, to the 6-Helix protein (Fig 5A), with an EC_50_ of 1.27 nM, and with increased binding to the 5-Helix protein (74 pM) (Fig 5B). Neither QA255.067 nor QA255.072 demonstrated detectable binding to either the 5-Helix or 6-Helix proteins, which is consistent with the mapping data showing they target the C-C’ loop region (Fig 5A and 5B).

We next tested whether any of the QA255 mAbs could mediate functional activity against cells coated with the 6-Helix bundle stump mimetic. Both mAbs QA255.006 and QA255.016 mediated ADCC against the 6-Helix target at levels significantly higher than control mAbs including QA255.157 that targets a CD4-induced epitope on gp120, and Fi6_v3, an influenza-specific antibody. Activity was ~ 2-fold lower than the positive control mouse mAb, NC-1, known to bind the stump mimetic [73]. Neither QA255.067 nor QA255.072 mediated ADCC in this assay, consistent with their lack of binding to this protein (Fig 5C).

### gp41 mAbs mediate killing of infected cells

**Fig 6:**
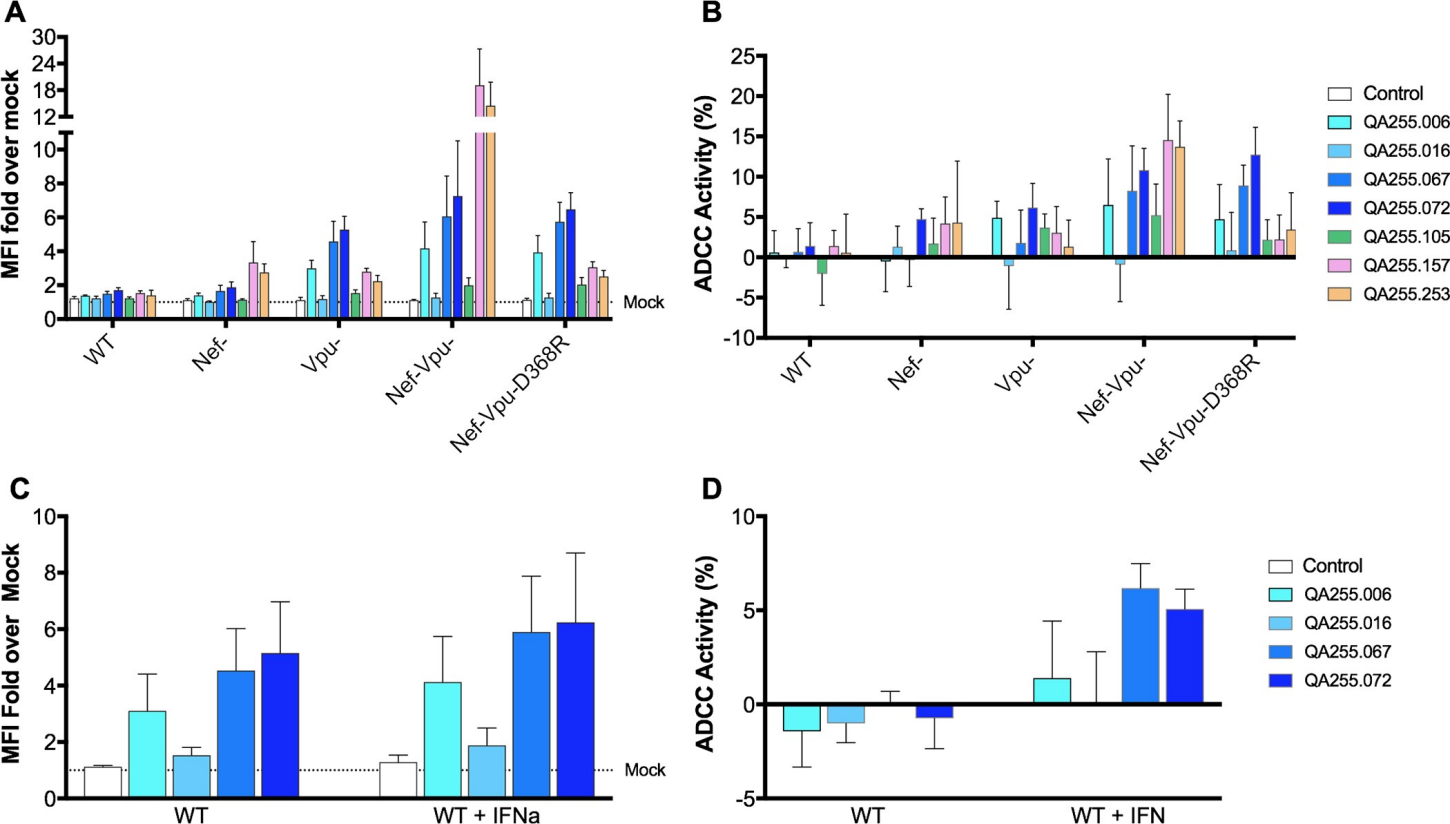
Infected cell recognition and ADCC susceptibility to Cluster I and Cluster II antibodies. (A and C) Binding or (B and D) ADCC activity was measured against cells infected with a wildtype NL4.3 virus construct expressing the ADA envelope (pNL43/ADA/WT), the construct with a deficient *nef* (pNL43/ADA/N-) or *vpu* gene (pNL43/ADA/U-), the construct with both *nef-* and *vpu-* deficient genes (pNL43/ADA/N-U-), or the construct with both deficient genes and containing the D368R mutation in the ADA envelope. In C and D cells were treated with IFNα as described in the Methods. Data represent the average +/- SD of 5 (A and B) and 4 (C and D) independent experiments.

All four Cluster I and Cluster II mAbs were tested for ADCC activity against cells infected with HIV-1, including viruses defective in *nef* and/or *vpu*, which leads to increased CD4 on the cell surface [74–76], enhanced exposure of CD4i epitopes [76–78] and increased Env density due to increased BST-2/Tetherin expression [76, 79]. A third virus with defective *nef* and *vpu* genes containing a mutation in the CD4-binding site (D368R) was tested to determine whether the mAbs were dependent on conformational changes induced by CD4 interaction [76, 78, 80]. The mAbs were tested with this virus panel for binding to the infected cells and ADCC activity, including with gp120-specific mAbs as controls. The gp120-specific mAbs, QA255.157 and QA255.253 [65], showed the highest level of binding to infected cells and corresponding high ADCC activity against cells infected with virus containing both defective *nef* and *vpu* genes (Fig 6A and 6B). As expected, this activity was impaired in cells infected with the D368R construct that eliminated CD4-Env binding and therefore exposure of CD4i epitopes [78, 80].

When the infected cell panel was tested against the four gp41 mAbs, all showed very little binding to cells infected with the wild type virus and this translated into no ADCC activity against these cells. QA255.006, QA255.067 and QA255.072 showed increased binding and ADCC activity against cells infected with the Vpu-deficient virus and the Vpu- and Nef-deficient viruses while QA255.016 showed barely detectable binding or ADCC activity against cells infected with all the viruses tested. This is consistent with Env accumulation at the surface of cells infected with Vpu-deficient viruses, as BST-2/Tetherin can mediate retention of viral particles [74, 75] on the cell surface. Accordingly, Interferon alpha (IFNα) treatment, which also enhances BST-2 retention of viral particles at the cell surface [75, 81], increased both recognition (Fig 6C) and ADCC susceptibility (Fig 6D) of cells infected with wild-type viruses. Finally, we observed no increase above wild type levels for cells infected with the Nef-deficient virus. For all of these mAbs, the presence of a mutation in the CD4 binding site (D368R) did not impact binding or ADCC activity, suggesting the epitopes recognized by these Abs are not dependent on structural changes that occur upon Env-membrane-bound CD4 interaction.

**Fig 7:**
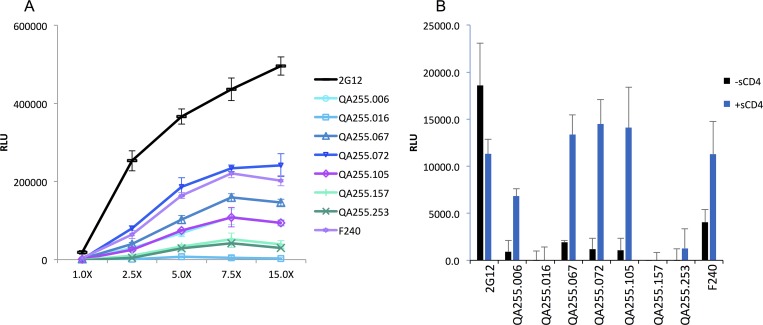
Cell based ELISA to detect Env recognition at the cell surface. (A) Binding to 293T cells transfected with an empty pcDNA3.1 plasmid or increasing concentrations of a plasmid expressing HIV-1_JRFLΔCT_ Env as described in the Methods. The key to the antibody tested is shown to the right. (B) Binding to cells pre-incubated in the presence or absence of sCD4 (10μg/mL for 1 hr at room temperature) before addition of the different anti-Env Abs. The concentration of plasmid expressing HIV Env used in the transfection corresponds to the 1X condition in panel A. For both panels, signals obtained with the empty pcDNA3.1 plasmid (negative control) were subtracted from signals obtained from Env-transfected cells. Results are presented as the average +/- SD of relative luminescence units (RLU). Results are representative from three independent experiments performed in quadruplicate.

Because the conditions that allowed detection of ADCC activity in the infected cell assay were when BST-2 levels promoted capture of viral particles, we could not determine if the gp41 mAbs are capable of binding to gp41 on the cell surface or if their binding reflects interaction with trapped viral particles, which would be consistent with the fact that they were isolated using viral particles as a bait [65]. To address this, we tested them in a cell-based ELISA assay where only Env is expressed at the cell surface [82]. QA255.006, QA255.067 and QA255.072 were able to bind Env at the cell surface, with higher binding detected at higher Env levels (as detected by 2G12, Fig 7A). Consistent with poor recognition of infected cells by QA255.016 (Fig 6), no binding for this Ab was observed in this system (Fig 7A). Thus, these data indicate that these gp41 mAbs do not require viral particles to interact with Env. Consistent with their ability to recognize a gp41 stump mimetic (Fig 5), we observed that sCD4-induced shedding as indicated by decreased 2G12 levels upon sCD4 addition, dramatically increased the ability of these mAbs to recognize Env (Fig 7B) further supporting the possibility that these mAbs recognize gp41 stumps. In addition, the same pattern is also seen for the anti-gp41 F240 mAb, which has also been suggested to recognize gp41 stumps [83]. How can this be reconciled with the observation that these mAbs do not more efficiently recognize cells infected with a virus deleted in Nef, which have higher levels of CD4 compared to cells infected with Nef containing virus [76, 77]? A potential explanation is that in cells infected with Nef- virus, CD4 interacts with Env in cis, thus occluding the access to the epitope, which is not the case when the Env is opened using sCD4. Supporting this, 8ANC195 does not efficiently recognize cells infected with Nef- virus [80] despite the fact that the structure of this mAb was obtained using a gp120 core stabilized with sCD4 [84].

## Discussion

There has been renewed interest in antibodies that mediate ADCC based on findings that ADCC antibody activity was associated with protection in the RV144 vaccine clinical trial [1] and in the setting of mother-to-child transmission [2, 3]. In addition, non-neutralizing ADCC antibodies have been associated with protection and delayed disease in NHP vaccine models and reduced viremia when passively infused prior to infection of NHP [12]. Here we describe four new gp41-specific ADCC mAbs that arose from four independent B cell lineages in one clade A infected individual. Two of these mAbs also recognize gp41 stumps and mediate ADCC against cells coated with stump mimetics. Importantly, these mAbs can mediate cell killing in multiple assays, including killing of productively infected T cells, the major source of virus in HIV infection. Notably, they mediate killing in infected cells exposed to IFN, a condition that is likely to be relevant to HIV infection *in vivo*.

The epitopes of these gp41 mAbs were mapped using both competition experiments and phage peptide display. Immunoprecipitation of a library of phage has the advantage of being able to interrogate a large number of peptides in a single well using deep sequencing to identify the specific peptides within the library that bind the mAbs. Our studies showed that QA255.067 and QA255.072 target the immunodominant C-C’ loop, which suggests they target cluster I. The phage display method allowed us to define a minimal epitope based on overlap in the sequences that bound these mAbs. These results suggest that there are subtle differences in the epitopes of these mAbs and also in these epitopes compared to a previously defined cluster I mAb, 240-D. Interestingly, the minimal epitope of QA255.067 excludes a variable residue at position 607, whereas this residue is included in the minimal epitopes of QA255.072 and variation at this residue appears to be tolerated by mAb QA255.072. Interestingly, longitudinal viruses cloned from QA255 over a more than four-year time period after infection demonstrated no variation in these residues, thus suggesting that ADCC antibody pressure is not sufficient to drive escape in this highly conserved region of gp41. We also mapped the epitope of a previously described mAb, 240-D to amino acids 596–605, which refined the epitope compared to the original 240-D epitope mapping study, which indicated the epitope was between 579–604 based on peptide binding studies [40]. Our results are also consistent with later studies examining binding to mutant forms of Env-gp160 protein, which suggested mutations at positions 596, 599 and 605 impact 240-D binding [70]. Overall, this analysis suggests that phage display could provide a high throughput tool for epitope mapping.

While the application of phage immunoprecipitation with deep sequencing was successful for mapping the epitopes of the cluster I mAbs, it was not for the mAbs with more complex, discontinuous epitopes. QA255.006 and QA255.016 share some properties of cluster II mAbs in that competition studies suggest their epitope includes the CHR. But competition experiments suggest that the target of these mAbs may also be discontinuous and include the fusion peptide proximal region and/or the NHR. These mAbs appear to enhance binding of the C-C’-loop, cluster I mAbs, as do other mAbs that target the CHR, such as 5F3. Interestingly, a mAb that bound a complex epitope on HIV gp41 was isolated from a clade B infected individual using VLPs to enrich for HIV-specific B cells suggesting these types of mAbs may be readily detected using VLPs as bait [85].

The QA255 derived gp41 mAbs all demonstrated measurable ADCC activity against cells coated with gp41, including the ectodomain expressed alone as well as within the context of the gp140 protein. Importantly, they also mediated killing of infected target cells, although the activity driven by mAb QA255.016 was very low. Poor ADCC activity by QA255.016 is consistent with the competition assay observations where QA255.006 was able to displace Bt-QA255.016 but QA255.016 was unable to displace BT-QA255.006. Activity was more readily detected with viruses lacking Vpu, presumably because the cells infected with *vpu*-deleted viruses have higher cell surface Env expression due to trapped viral particles [74–76, 81, 86, 87]. Accordingly, stimulation with IFNα, known to induce retention of viral particles at the surface of infected cells [75, 81], increased recognition and ADCC activity of these Abs. The activity was not dependent on Env-CD4 interaction at the cell surface because it was not increased compared to wild type virus when a *nef*-deleted virus was used. CD4i epitopes are a common target of non-neutralizing gp120-specific mAbs that mediate ADCC and can result in killing of bystander cells that have shed gp120 on their surface [79, 88]. As such, the gp41 mAbs would be predicted to have fewer off-target effects that result in this undesirable killing of HIV negative cells.

In the pre-hairpin conformation, both the NHR and CHR of gp41 are exposed. As such, this pre-hairpin fusion intermediate has been identified as a target for fusion inhibitors and as a vaccination target. In the post-fusion conformation, the fusion peptide and the transmembrane anchor are irreversibly brought together into a stable 6-helical bundle [89]. Thus, this structural form of HIV gp41 represents a potential target for HIV-specific antibodies. The two mAbs described here that include the CHR within their epitope target are capable of binding to the 6-helix structure and mediating killing of target cells coated with this form of the protein; they also mediate killing of infected cells. Importantly, these two mAbs, along with the more potent of the two mAbs targeting the C-C’ loop bound to Env expressed on the cell surface, with much higher levels of binding under conditions that induced gp120 shedding. Collectively, these findings suggest that these mAbs could participate in the elimination of infected cells that have shed gp120, which can expose the gp41 stump [26].

The presence of gp41 antibodies that mediate ADCC in plasma has long been appreciated [27, 90]. Many previous studies showed that gp41-directed antibody responses are generally common in HIV-infection, including responses to epitopes that are similar to those of the mAbs studied here [36, 40, 91–93]. Despite the common nature of gp41 plasma antibody responses, relatively few gp41-specific mAbs that mediate ADCC have been described. Many of the previously characterized mAbs are IgG2 [28, 31], an isotype which primarily mediates killing via macrophages and neutrophils through the FcγRIIa. IgG2 also has very low affinity for the Fc receptor most important for NK-cell mediated ADCC activity, FcRγIIIa [94]. The gp41 ADCC mAbs described here were encoded as IgG1, which can interact with a range of FcγRs. IgG1 is also the most abundant antibody and thus a major driver of the ADCC response. In addition to ADCC, gp41-specific mAbs have been shown to block transcytosis of virus [47, 53] and to inhibit virus infection in dendritic cells and macrophages by mechanisms that likely involve effector functions [44, 46]. Moreover, gp41-specific IgA activity has been linked to resistance from infection in highly exposed seronegative individuals [57]. Thus overall, gp41-specific antibodies may make unique contributions to decreasing HIV transmission and pathogenesis. In this regard, the effect of IFN on ADCC activity observed here may be particularly relevant given that IFN is an early antiviral response.

Four of the twelve HIV-specific mAbs isolated from a clade A infected individual targeted gp41 and they were all derived from independent lineages, even though there were two pairs of mAbs, with each pair targeting similar epitopes. This suggests that gp41-specific mAbs that mediate ADCC may be a common response during chronic HIV infection and the antibodies isolated here will be useful as reagents for testing this hypothesis. These ADCC mAbs from 914 days post infection showed relatively low SHM (VH: 6.5–12.9%; VL/VK: 3.7–8.8% NT) (Table 1) compared to broadly neutralizing mAbs. Two of the four gp41-specific mAbs described here, QA255.067 and QA255.072, utilize gene IGVH1-69 (Table 1), which is common for cluster I-directed mAbs [95].

One of the challenges in eliciting a protective response against HIV, particularly for eliciting protective neutralizing antibodies, is the diversity of the Env antigen. To date, the gp41-specific mAbs identified after HIV vaccination have tended to be polyreactive and not able to mediate HIV-specific ADCC activity [96]. ADCC Abs tend to target conserved epitopes and show breadth [12, 65, 97–100]. In terms of breadth of the gp41 protein, in particular the ectodomain, gp41 is a particularly attractive target because it is more conserved than most gp120 regions targeted by bnAbs [25]. Thus, the new ADCC Abs described here, that target conserved regions in gp41 and mediate killing of HIV infected cells may provide insight into the features of antibodies that can mediate broad protection against HIV infection.

## Methods

### QA255 antibody synthesis

Antibodies from QA255 were originally isolated and cloned as described previously [65]. In brief, paired heavy and light chain DNA clones were co-transfected in equal ratios into 293F cells (293 Freestyle cells; Thermo Fisher; 1x10^6^ cells/1 μg of total DNA) with a 16:1:1 (OptiPRO Serum-Free Medium:293Max:DNA, Thermo Fisher) ratio. Antibodies were harvested after 72 h and purified using Protein G resin in hand-packed, gravity flow columns (Pierce). Antibody concentration was determined using protein absorbance at 280 nM (Nanodrop).

### Binding Antibody Multiplex Assay (BAMA)

The BAMA was conducted as described [1, 32, 98, 99] to measure IgG binding to a panel of HIV antigens. Prior to performing the BAMA, antigens were covalently conjugated to carboxylated fluorescent beads (Luminex) as described previously [32, 100]. Antigen-conjugated beads were stored in PBS (Gibco) containing 0.1% bovine serum albumin (BSA; Sigma-Aldrich), 0.02% Tween (Sigma-Aldrich), and 0.05% sodium azide (Sigma-Aldrich) at the optimal temperature for the unconjugated antigen for up to 1 year. Antigens included in the assay were monomeric gp120 proteins BG505.W6M.C2.T332N (clade A), BL035.W6M.ENV.C1 (clade A/D recombinant), SF162 (clade B), ZM109F.PB4 (clade C), C2-94UG114 (clade D), and SIV/mac239; clade A BG505 SOSIP Env trimer (courtesy of Marit van Gils, Rogier Sanders and John Moore) [66]; resurfaced Env core protein (RSC3) and CD4-binding site defective mutant (RSC3 Δ371I) (construct obtained from NIH AIDS Reagent Program, Division of AIDS, NIAID, NIH from Drs. Zhi-Yong Yang, Peter Kwong, Gary Nabel) and produced as described in [101]; clade C 2J9C-ZM53_V1V2 and 1FD6-Fc-ZM109_V1V2 scaffolded peptides [102]; V3 consensus peptides ConA1 (CTRPNNNTRKSIRIGPGQAFYATGDIIGDIRQAHC) and ConB (CTRPNNNTRKSIHIGPGRAFYTTGEIIGDIRQAHC) (Genscript); and two gp41 antigens: clade B MN gp41 monomer (NIH AIDS Reagent Program, Division of AIDS, NIAID, NIH from ImmunoDX, LLC) and clade C ectodomain ZA.1197/MB (Immune Technology Corp). BG505 gp120 was produced by transient transfection of 293F cells (Thermo Fisher) followed by *Galanthus nivalis* lectin purification (Vector Laboratories) as described previously [103]. All other gp120 proteins were purchased from Immune Tech. Positive controls included VRC01, PG9, PGT121, 4E10, 50–69, and 246-D. VRC01, PG9 and PGT121 were all produced as described above and 4E10, 50–69, and 246-D obtained from the NIH AIDS Reagent Program, Division of AIDS, NIAID, NIH (4E10 from Polymun Scientific, and 50–69 and 246-D from Dr. Susan Zolla-Pazner). Negative controls included both HIV-negative plasma and mock conjugated beads. Binding is measured as the mean fluorescence intensity (MFI) and averaged across duplicate wells. Results are reported as fold change over binding by HIV-negative plasma.

### gp41 binding ELISA

The gp41 binding ELISA was adapted from [65]. In brief, Immunolon 2-HB plates were coated with 100 μL of MN gp41 (NIH AIDS Reagent Program, Division of AIDS, NIAID, NIH from ImmunoDX, LLC.) or ZA.1197 (Immune Technology Corp) at 0.5 μg/mL in 0.1 M sodium bicarbonate coating buffer (pH 7.4) overnight at 4°C. Plates were rinsed 4–5 times using PBS-0.05% Tween wash buffer. Plates were blocked with 10% non-fat dry milk (NFDM) diluted into wash buffer for at least 1 h. After removing the blocking buffer, 100 μL of primary mAb diluted in blocking buffer was added and incubated at 37°C for 1 hr. Plates were washed a second time and 100 μL of anti-IgG-HRP (Sigma-Aldrich) diluted 1:2500 in blocking buffer was added and incubated at room temperature for 1 hr. Plates were washed and 50 μL Ultra-TMB (Thermo Scientific) substrate added to each well and incubated at room temperature for 10 min. This reaction was stopped by adding 50 μL of 0.1 M H2SO4 (Sigma-Aldrich) and the absorbance was read at 450 nM optical density within 30 min. The endpoint titers for all antibodies were defined as the average Ab concentration with binding greater than 2-fold of the negative control, Influenza-specific mAb Fi6_v3 (courtesy of Jesse Bloom and Kelly Lee).

### 6-helix and 5-helix protein purification and ELISA

Gp41 mimetics 6-helix and 5-helix were expressed in *E*. *coli* and purified as described previously [71]. The 6-helix and 5-helix constructs were modified (K68C) to allow biotinylation using maleimide chemistry. Briefly, the proteins were reduced in degassed conditions with 5mM TCEP, then incubated with 5 molar excess of EZ-link Maleimide-PEG_11_-biotin (Thermo Fisher) reagent for 1 hr at room temperature. Unreacted biotinylation reagent was removed using a PD-10 desalting column according to manufacturer’s instructions (GE Healthcare). Biotinylation efficiency was determined using HABA reagent (Pierce).

For ELISA assays, 96-well plates (Nunc Maxisorp^TM^ flat-bottom, Thermo Fisher Scientific) were coated with 5 μg/mL streptavidin (in 50 mM sodium bicarbonate pH 8.75) for at least 1 hr, before the addition of 5 μg/mL biotinylated 6-helix or 5-helix. Following coating with antigens, the plates were washed three times with 300 μL 1xPBST and blocked with 300 μL of 1xPBST with 0.5% BSA for at least one hour. Following blocking, antibodies were added in serial 10-fold dilutions starting at 75 μg/mL for at least 1hr. The plates were then washed 3x with 300 μL of 1xPBST and an anti-human IgG HRP secondary antibody (Thermo Fisher) was added for 1 hr at room temperature. The plates were then washed 6x with 300 μL of 1xPBST and developed using 1-Step^TM^ Turbo TMB ELISA substrate solution (Thermo Fisher Scientific) for 6 mins and quenched using 2M H_2_SO_4_. The readout of this colormetric assay was determined using a 96 well plate reader (Biotek), and the intensity of the absorbance at 450 nm was normalized for the path length. Finally, these resulting values were baseline subtracted (subtracting the average of the background signal from secondary antibody only control wells). EC50s were obtained from fitting values to a sigmoidal curve in GraphPad Prism v7.0c.

MAb D5, which binds to the highly conserved hydrophobic pocket on the NHR [104], was obtained through the NIH AIDS Reagent Program, Division of AIDS, NIAID, NIH from Dr. Danilo Casimiro.

### Rapid and Fluorometric ADCC (RF-ADCC) assay

The RF-ADCC assay was performed as described [2, 3, 65, 67]. In short, CEM-NKr cells (AIDS Research and Reference Reagent Program, NIAID, NIH from Dr. Alexandra Trkola) were double labeled with PKH26-cell membrane dye (Sigma-Aldrich) and a cytoplasmic-staining CSFE dye (Vybrant CFDA SE Cell Tracer Kit, Life Technologies). The double-labeled cells were coated with either clade A gp140 (Q461.e2) [105], MN gp41 (NIH AIDS Reagent Program, Division of AIDS, NIAID, NIH from ImmunoDX, LLC.), or the 6-helix gp41 mimetic for 1 hr at room temperature at a ratio of 0.15–1.5 μg protein (1 μg/μL):1 x 10^5^ double-stained target cells. Coated targets were washed once with complete RMPI media (Gibco) supplemented with 10% FBS (Gibco), 4.0mM Glutamax (Gibco) and 1% antibiotic-antimycotic (Life Technologies). Monoclonal antibodies were diluted in complete RPMI media to a concentration of 50–500 ng/mL, depending on the antigen and mixed with 5 x 10^3^ coated target cells for 10 min at room temperature. PBMCs (peripheral blood mononuclear cells; Bloodworks Northwest) from an HIV-negative donor were then added at a ratio of 50 effector cells per target cell. The coated target cells, antibodies, and effector cells were co-cultured for 4 hr at 37°C then fixed in 150 μL 1% paraformaldehyde (Affymetrix). Cells were acquired by flow cytometry (LSR II, BD) and ADCC activity defined as the percent of PE+, FITC- cells with background subtracted where background (antibody-mediated killing of uncoated cells) was between 3–5% as analyzed using FlowJo software (Tree Star). The data were plotted with percent ADCC activity on the y-axis and respective mAb on the x-axis (Graphpad Prism v7.0c).

### Competition ELISAs

Antibodies selected for the competition ELISA experiments were all obtained from the NIH AIDS Reagent Program, Division of AIDS, NIAID and included: 5F3 and 2F5 (provided by H. Katinger); 167-D, 240-D, 50–69, and 246-D (provided by S. Zolla-Pazner); F240 (provided by M. Posner and L. Cavacini); D5 (provided by D. Casimiro).

Immunolon 2-HB plates were coated with MN gp41 as described above. Competitor antibodies were added first at a concentration of 10 μg /mL to gp41-coated plates and incubated for 15 min at 37°C. Biotinylated (BT; Thermo Fisher Scientific) QA255 antibodies were added next without washing and the competitor/ BT-antibody mixture were incubated together for 45 min at 37°C. Limiting concentrations for each BT mAb were pre-determined as follows: BT-QA255.006 at 1.25 μg/mL, BT-QA255.016 at 10 μg/mL, BT-QA255.067 and BT-QA255.072 both at 0.625 μg/mL. Plates were then washed thoroughly and HRP-conjugated Streptavidin diluted in wash buffer (1:1000) added and incubated for at least 45 min. After washing, Ultra TMB-substrate and 0.1 M H_2_SO_4_ were added as previously described. Relative BT-mAb binding was calculated by dividing each BT-mAb binding in the presence of each competitor antibody by the average of the same BT-mAb binding in the presence of blocking buffer.

### Phage display immunoprecipitation-sequencing

To identify precise epitopes of antibodies in this study, we utilized an approach that couples phage immunoprecipitation and highly-multiplexed sequencing [69]. We used a phage-display library that contains several full-length HIV sequences from each clade, including consensus sequences from Clades A, B, C, and D (LANL), Q23 (AF004855.1), BF520.W14M.C2 (KX168094), BG505.W6.C2 (DQ208458), and Env sequences from QA013.70I.Env.H1 (FJ866134), QA013.385M.Env.R3 (FJ396015), QB850.73P.C14 (MK412338), QB850.632P.B10 (MK412339), Q461.D1 (AF407155), and QC406.F3 (FJ866133).

To generate the library, 39-amino acid sequences were generated that tiled over the coding sequences of viral genomes of interest with 20-amino acid overlap. These protein sequences were reverse translated to DNA sequences and codon-optimized for expression in *E*. *coli*. Synonymous mutations were introduced to avoid EcoRI and HindIII restriction sites that were used in subsequent cloning steps. Adapter sequences (5’: AGGAATTCTACGCTGAGT and 3’: TGATAGCAAGCTTGCC) were added and the library was ordered on a releasable DNA microarray (Twist Biosciences). The library was then PCR amplified using our T7F (AATGATACGGCAGGAATTCTACGCTGAGT) and T7R (CGATCAGCAGAGGCAAGCTTGCTATCA) primers, digested with EcoRI and HindIII, cloned into the T7Select^®^ 10-3b Vector, and packaged into T7 phage and amplified according to the manufacturer’s protocol (EMD Millipore).

Phage immunoprecipitation was performed as previously described [69]. 96-deep-well plates (CoStar) were blocked with 3% BSA in TBST (Tris-buffered saline-Tween) by placing on a rotator overnight at 4°C. 1 mL of amplified phage at 2x10^5^-fold representation (1.2x10^9^ pfu/mL for a library of 5.8x10^3^ phage) was added to each well, followed by either 2ng or 10ng of purified anti-gp41 monoclonal antibody. Each concentration of monoclonal antibody was tested in technical replicate. Phage-antibody complexes were formed by rotating the plate at 4°C for 20 hours. To immunoprecipitate phage-antibody complexes, 40μL of a 1:1 mix of protein A and protein G Dynabeads (Invitrogen) was added to each well and rotated at 4°C for 4 hours. After this incubation, a magnetic plate was used to isolate the beads and perform 3 washes with 400μL of wash buffer (50mM Tris-HCl, pH 7.5, 150mM NaCl, 0.1% NP-40). The beads were resuspended in 40μL of water and isolated phage were lysed by incubating at 95°C for 10 mins. Phage that did not undergo immunoprecipitation (‘input’) were also lysed to determine the starting frequencies of each phage clone in the library.

Isolated phage DNA was then prepared for highly-multiplex sequencing by performing two rounds of PCR with Q5 High-Fidelity DNA polymerase (New England Biolabs) to add Illumina adapters and barcodes according to the manufacturer’s suggested protocol (NEB). The first-round PCR was performed with primers R1_F (TCGTCGGCAGCGTCTCCAGTCAGGTGTGATGCTC) and R1_R (GTGGGCTCGGAGATGTGTATAAGAGACAGCAAGACCCGTTTAGAGGCCC). 1μL of purified first-round product was added to the second-round PCR with unique dual-indexed primers R2_F (AATGATACGGCGACCACCGAGATCTACACxxxxxxxxTCGTCGGCAGCGTCTCCAGTC) and R2_R (CAAGCAGAAGACGGCATACGAGATxxxxxxxxGTCTCGTGGGCTCGGAGATGTGTATAAGAGACAG). In these primer sequences, “xxxxxxxx” corresponds to a unique 8-nt indexing sequence. Second-round PCR products were quantified in each sample using Quant-iT PicoGreen according to the manufacturer’s suggested protocol (Thermo Fisher). Equimolar quantities of each sample were then pooled, gel isolated, and submitted for Illumina sequencing on a MiSeq, where 60,000–1,100,000 reads were obtained for each sample.

Bioinformatics analyses of the sequencing data was performed as previously described [69]. In brief, a zero-inflated generalized Poisson significant-enrichment assignment algorithm was used to generate a–log_10_(p-value) for enrichment of each clone across all samples. A reproducibility threshold was established to call ‘hits’ in technical replicate pairs by first calculating the log_10_(-log_10_(p-value)) for each clone in Replicate 1. We then surveyed these values in Replicate 2 by using a sliding window of width 0.01 from -2 to the maximum log_10_(-log_10_(p-value)) value in Replicate 1. For all clones that fell within each window, the median and median absolute deviation of log_10_(-log_10_(p-values)) in Replicate 2 were calculated and plotted against the window location. The reproducibility threshold was set as the window location where the median was greater than the median absolute deviation. The distribution of the threshold–log_10_(p-values) was centered around a median of 2.2. In sum, we called a phage clone a ‘hit’ if the–log_10_(p value) was at least 2.2 in both replicates. Beads-only samples, which serve as a negative control for non-specific binding of phage, were used to identify and eliminate background hits. Peptides called as hits were aligned using Clustal Omega. The shortest amino acid sequence present in all of the hits was what we defined as the “minimal epitope” of an antibody. Of note, peptides were tiled as described above.

### QA255 envelope cloning and sequencing

Methods describing amplification and characterization of envelope clones from PMBC DNA from 189, 560, 662 and 1729 days post-infection were previously described [106]. Envelope clones from 21 days post-infection were generated from plasma RNA using similar methods. In both cases, a limiting dilution PCR strategy was used to amplify single genome envelope sequences.

### Flow cytometry analysis of cell-surface staining and ADCC

For cell surface staining, infected or mock-infected CEM.NKr (CEM cells resistant to Natural Killer cells killing, from Dr. David T Evans [4]) were incubated for 30 min at room temperature 48 h post-infection with 5 μg/ml of each tested antibody in PBS. Cells were then washed twice with PBS and stained with 1 μg/ml of goat anti-human antibody (Alexa Fluor-647, Invitrogen) for 15 min in PBS. After two more PBS washing, cells were fixed in a 2% PBS-formaldehyde solution. ADCC was performed with a previously described assay [76]. Briefly, CEM.NKr infected cells were stained with viability (AquaVivid; Invitrogen) and cellular (cell proliferation dye eFluor670; eBiosciences) markers and used as target cells. Effector PBMCs, stained with another cellular marker (cell proliferation dye eFluor450; eBiosciences), were then mixed at an effector/target (E/T) ratio of 10:1 in 96-well V-bottom plates (Corning); 5 μg/ml of the desired Ab was added to appropriate wells. Co-cultures were centrifuged for 1 min at 300 g and incubated at 37°C for 5–6 hr before being fixed in a 2% PBS-formaldehyde solution containing 5x10^4^/ml flow cytometry particles (AccuCount Blank Particles, 5.3 μm; Spherotech). IFN-α (PBL Assay Science) was reconstituted in RPMI-1640 complete medium at 1x10^7^ U/mL, aliquoted, and stored at −80°C. IFN-α was then added to the cells at 1000 U/mL 24h post-infection, 24h before cell-surface staining or ADCC assays. Samples were analyzed on an LSRII cytometer (BD Biosciences) and acquisition was set to acquire 1000 particles, which allows the calculation of relative cell counts. Data analysis was performed using FlowJo vX.0.7 (Tree Star). The percentage of cytotoxicity was calculated with the following formula: ((relative count of GFP^+^ cells in Targets plus Effectors)—(relative count of GFP^+^ cells in Targets plus Effectors plus antibodies)) / relative count of GFP^+^ cells in Targets.

### Cell-based ELISA

Detection of trimeric Env at the surface of HOS (human osteosarcoma, ATCC) cells was performed by cell-based ELISA, as previously described [82, 107]. Briefly, HOS cells were seeded in T-75 flasks (3 × 10^6^ cells per flask) and transfected the next day with either 3.0 (1x), 7.5, 15.0, 22.5 or 45.0 μg per flask with the empty pcDNA3.1 vector or expressing the codon-optimized HIV-1_JRFL_ envelope glycoproteins with a truncation at position Gly 711 in the cytoplasmic tail (ΔCT), enhancing cell-surface expression. Cells were transfected with the standard polyethylenimine (PEI, Polyscience Inc, PA, USA) transfection method. Twenty-four hours after transfection, cells were plated in 384-wells plates (2 × 104 cells per well) and one day later, cells were incubated in Blocking Buffer (Washing Buffer [25 mM Tris, ph 7.5, 1.8 mM CaCl_2_, 1.0 mM MgCl_2_, pH 7.5 and 140 mM NaCl] supplemented with 10 mg/ml non-fat dry milk and 5 mM Tris pH 8.0) for 30 minutes and then pre-incubated or not for 1 h with soluble CD4 (sCD4) (10 μg/ml) diluted in Blocking Buffer at room temperature. Cells were incubated with the anti-HIV-1 Env monoclonal antibodies (2G12, QA255.006, QA255.016, QA255.067, QA255.072, QA255.105, QA255.157, QA255.253, F240) in absence or presence of sCD4 (10 μg/ml) in blocking buffer. Cells were washed five times with Blocking Buffer and five times with Washing Buffer. A horseradish peroxidase (HRP) conjugated antibody specific for the Fc region of human IgG (Pierce) was then incubated with the samples for 45 minutes. Cells were washed again five times with Blocking Buffer and five times with Washing Buffer. All incubations were done at room temperature. 20 μl of a 1:1 mix of Western Lightning oxidizing and enhanced luminol reagents (Perkin Elmer Life Sciences) was added to each well. Chemiluminescence signal was acquired for 1 sec/well with the LB 941 TriStar luminometer (Berthold Technologies).

## Supporting information

S1 Fig gp41-specific QA255 mAbs mediate ADCC activity with PBMCs from second donor. (TIFF)

S2 Fig Competitor mAbs of known epitope specificity bind MN gp41 protein. (TIFF)

S3 Fig Competition binding assay with mAbs of known epitope specificity using gp41 ectodomain ZA.1197. Binding of biotinylated variants A) QA255.006 and B) QA255.016 to gp41 ectodomain protein ZA.1197. Binding was assessed in competition with the panel of mAbs on the right side of the figure with defined epitope specificity.
(TIFF)

S4 Fig Alignment of QA255 HIV envelope sequences to evaluate gp41-mAb-specific escape. Alignment of the ectodomain of gp41 for 28 QA255 homologous Env amino acid sequences. The epitope of QA255.067 and QA255.072 defined in Fig 4 and the epitope of mAbs that competed with QA255.006 and QA255.016 (5F3, 167-D; Fig 3) are marked, as are the fusion peptide, NHR and CHR.
(TIFF)
