# Supplementary figures and images for: Identification of HIV gp41-specific antibodies that mediate killing of infected cells
Source: PLoS Pathog. 2019 Feb 19;15(2):e1007572. doi: 10.1371/journal.ppat.1007572 (PMC6396944; doi:10.1371/journal.ppat.1007572)

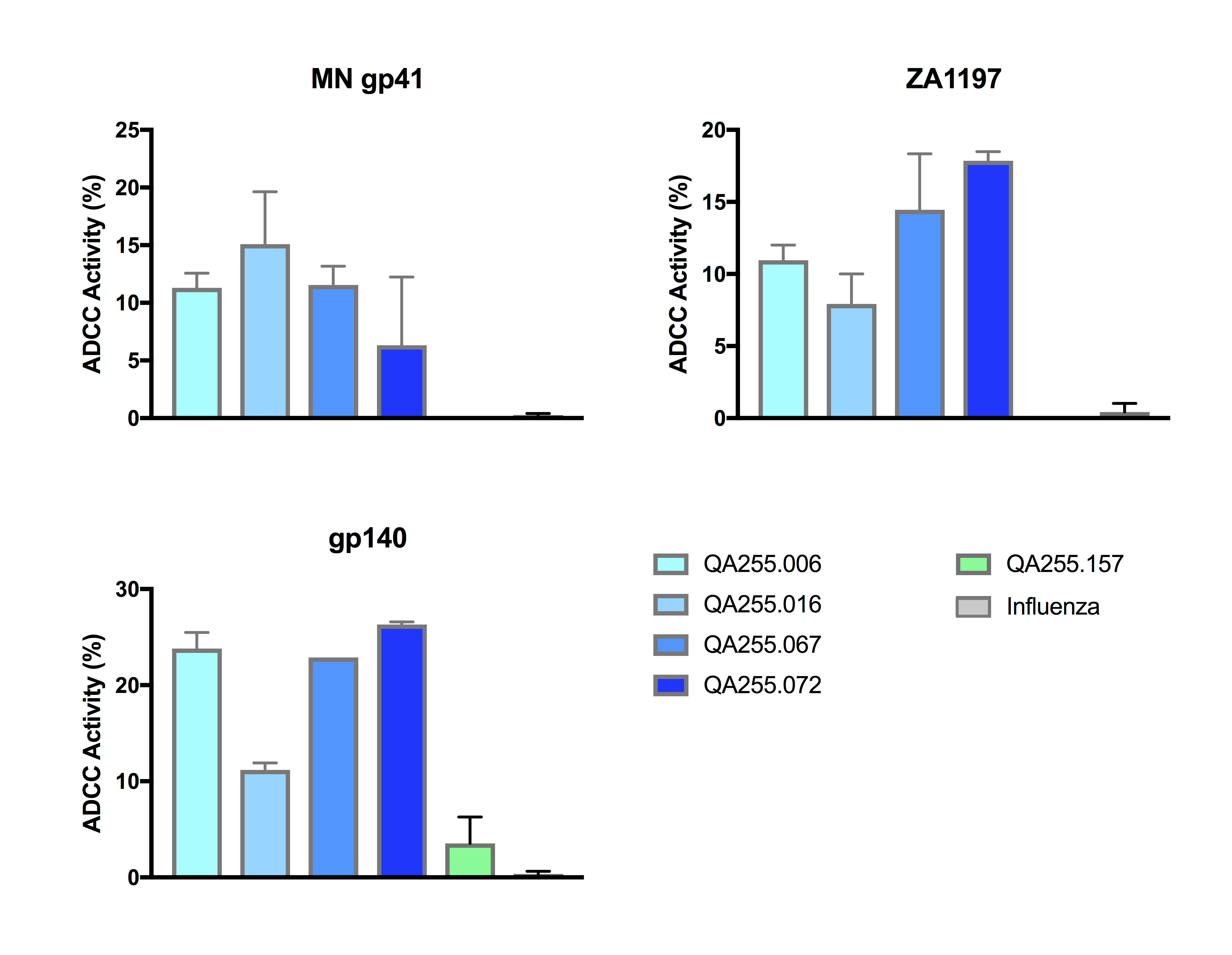

Supplement: S1 Fig — (TIFF) [file ppat.1007572.s001.tiff]

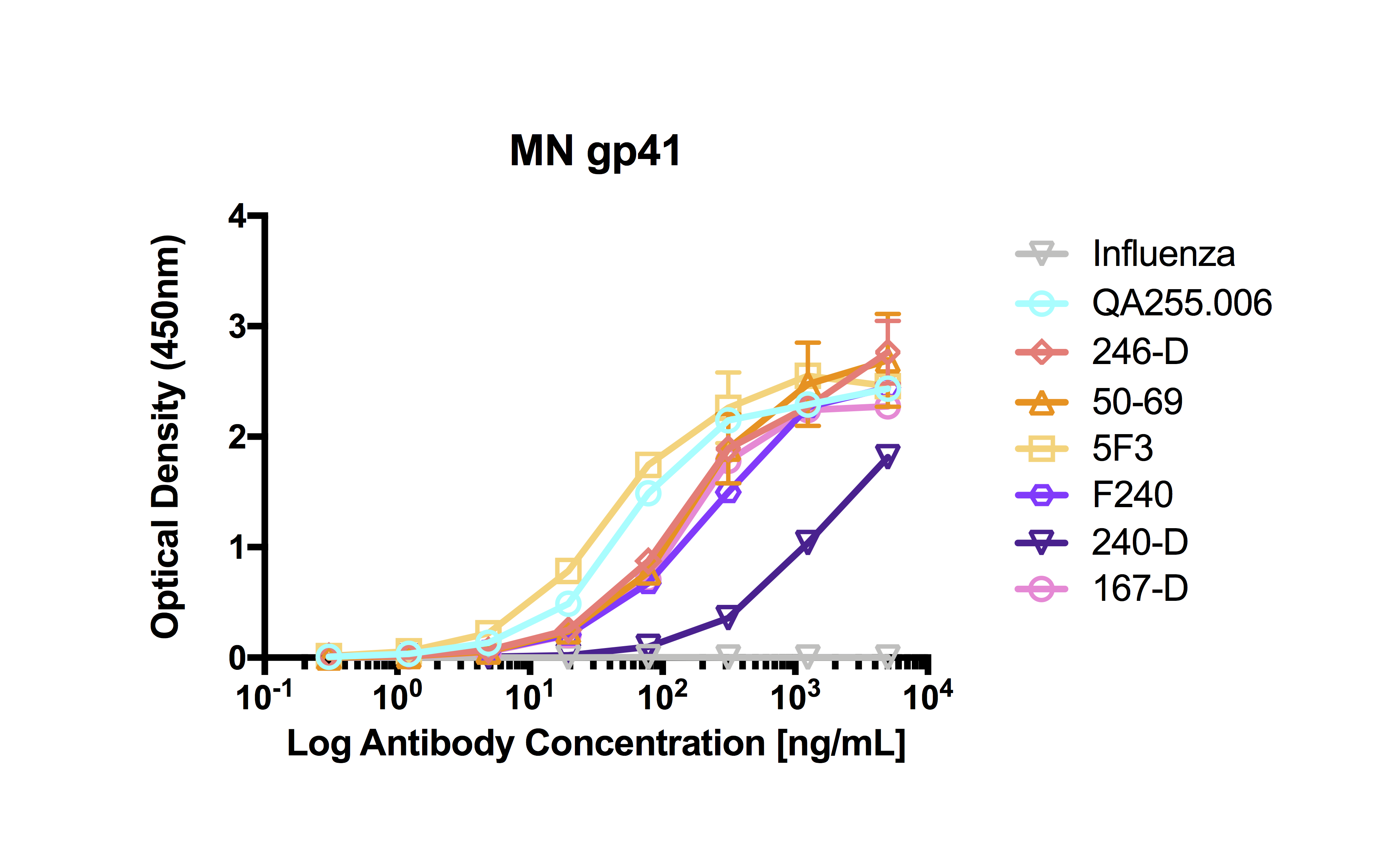

Supplement: S2 Fig — (TIFF) [file ppat.1007572.s002.tiff]

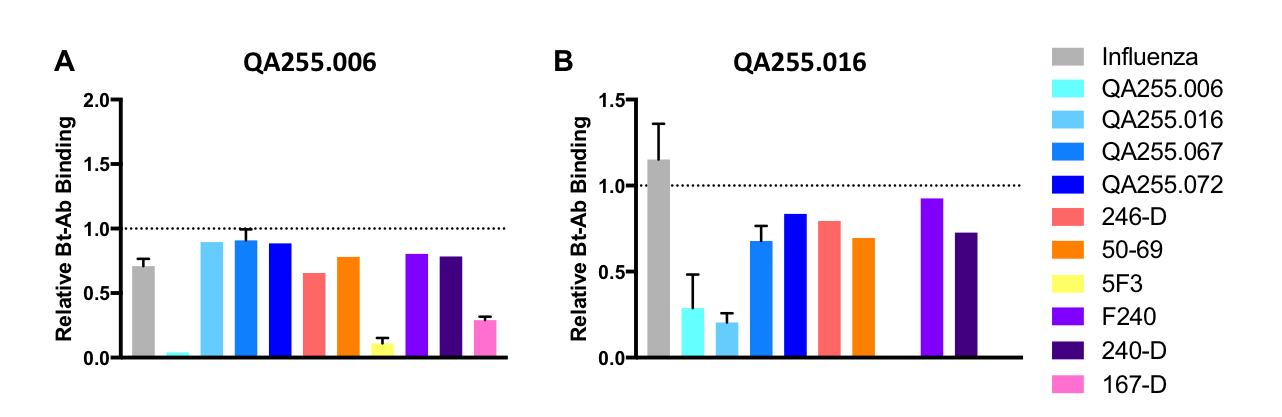

Supplement: S3 Fig — Binding of biotinylated variants A) QA255.006 and B) QA255.016 to gp41 ectodomain protein ZA.1197. Binding was assessed in competition with the panel of mAbs on the right side of the figure with defined epitope specificity. (TIFF) [file ppat.1007572.s003.tiff]

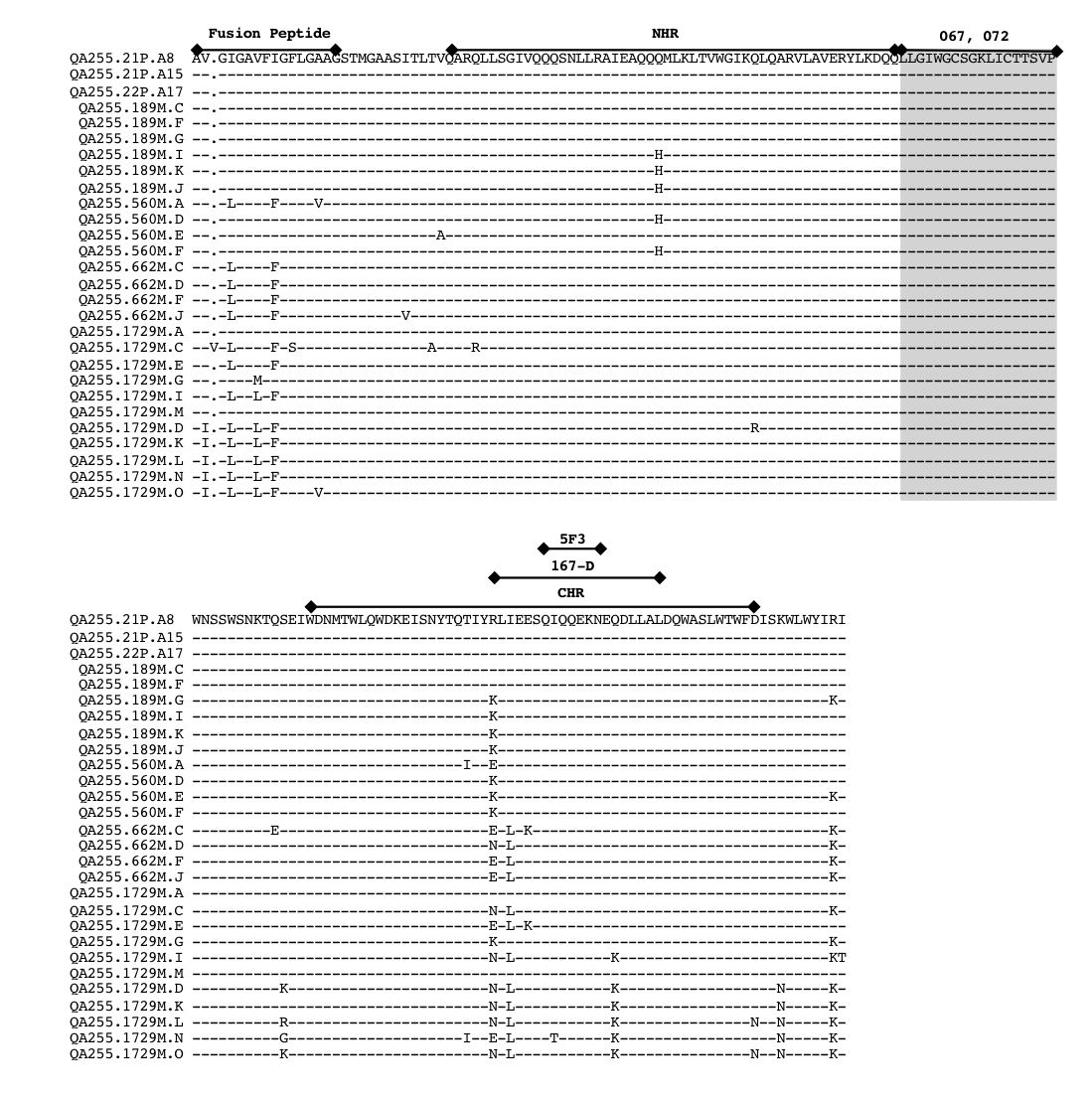

Supplement: S4 Fig — Alignment of the ectodomain of gp41 for 28 QA255 homologous Env amino acid sequences. The epitope of QA255.067 and QA255.072 defined in Fig 4 and the epitope of mAbs that competed with QA255.006 and QA255.016 (5F3, 167-D; Fig 3) are marked, as are the fusion peptide, NHR and CHR. (TIFF) [file ppat.1007572.s004.tiff]
